# Oral Capecitabine Achieves Response in Metastatic Eccrine Carcinoma

**DOI:** 10.1155/2018/7127048

**Published:** 2018-03-01

**Authors:** Kristian Larson, Hani M. Babiker, Andrew Kovoor, Joy Liau, Jordan Eldersveld, Emad Elquza

**Affiliations:** ^1^Early Phase Clinical Trial Program, The University of Arizona Cancer Center, 1515 N. Campbell Ave., Tucson, AZ 85724, USA; ^2^Translational Genomics Research Institute (TGen), 445 N. 5th Street, Phoenix, AZ 85004, USA; ^3^Division of Hematology and Oncology, University of Arizona College of Medicine, 1515 N. Campbell Ave., Tucson, AZ 85724, USA; ^4^Department of Medical Imaging, University of Arizona, 1501 N. Campbell Ave., Tucson, AZ 85712, USA; ^5^Department of Pathology, University of Arizona, 1501 N. Campbell Ave., Tucson, AZ 85712, USA

## Abstract

The low prevalence rate and limited literature on eccrine carcinoma (EC) pose a challenge to properly diagnosing and treating this rare malignancy. EC lesions tend to present similarly to other cutaneous neoplasms and dermatitis-like conditions. Efficacious treatment guidelines have not been established for patients diagnosed with EC, and few treatment regimens have demonstrated clinical benefit. Due to the high metastatic potential of EC, recognizing the clinical presentation, properly diagnosing, and utilizing beneficial treatment options are important for managing this disease. We report a case of a 66-year-old female who presented with lesions that her primary care provider misdiagnosed as basal cell carcinoma. The disease responded poorly to taxane- and platinum-based chemotherapies as well as an isolated limb perfusion of an alkylating agent. However, continuous dosing of oral capecitabine achieved an 18-month period of progression free survival (PFS) and ameliorated quality of life. We wish to highlight this rare disease and discuss presentation, diagnosis, and management as it is most often misdiagnosed leading to advanced metastatic disease when patients present to the oncologist. In addition, it is crucial to study and report potentially efficacious regimens considering the lack of clinical trials in this disease.

## 1. Introduction

Eccrine carcinoma (EC) is an appendageal cancer that originates from the eccrine sweat gland and accounts for an estimated 0.005–0.01% of diagnosed cutaneous malignancies [1]. Histopathologies closely associated with EC include hidroadenocarcinoma, spiradenocarcinoma, and porocarcinoma [1–3]. A consistent clinical presentation of these lesions has not been identified; other authors have reported brown, bluish, and erythematous lesions which may appear papular or ulcerative [1, 4, 5]. Proper recognition and diagnosis of EC prove to be difficult due to its infrequency and similar presentation to basal and squamous cell carcinoma, amelanotic melanoma, seborrheic keratosis, cutaneous lymphoma, and verruca vulgaris [1]. EC lesions are commonly reported in the lower extremities (35%), head and neck (24%), and upper extremities (14%) [1]. Immunohistochemical (IHC) stains and serum cancer markers have also been inconsistently reported, which include but are not limited to carcinoembryonic antigen, epithelial membrane antigen, estrogen receptors, progesterone receptors, cytokeratin 7, and pancytokeratins [2, 6]. Thus, recognizing the pleomorphic features of basaloid cells within the epidermis that infiltrate into the ductal papillary structures of the dermis help diagnose EC [2].

A limited understanding of EC in addition to unestablished treatment guidelines contributes to a relative mortality rate of 80% and a 10-year disease survival rate of 9% when metastatic in stage [1, 7]. Based on the current literature, there seems to be a clinical consensus that wide surgical excision with the goal of clear margins is the most effective treatment for locoregional EC [8]. However, this option is not optimal for metastatic cases [9]. Other cases of metastatic EC have illustrated resistance to radiotherapy as well as a variety of chemotherapies [9]. We describe a case of a patient who continued to progress during treatments with carboplatin, paclitaxel, melphalan, actinomycin D, and radiotherapy but achieved 18 months of progression free survival (PFS) and an improved quality of life with oral capecitabine despite her diffusely metastatic disease.

## 2. Case Description

A 66-year-old female with a history of ongoing thrombocytosis (platelets at 724 × 10^3^/uL, range: 150–450 × 10^3^/uL), emphysema and coronary artery disease visited her primary care physician (PCP) with complaints about pruritic “bumps” on her left calf in 2007. Her PCP noted that the lesions presented as erythematous, 2-3 mm in diameter, and papular. The PCP diagnosed these neoplasms as basal cell carcinoma without further diagnostic testing or intervention. The patient returned in early 2009 with enlarged lesions, increased pruritus, and ulceration. The patient was referred to dermatology and oncology.

**Figure 1:**
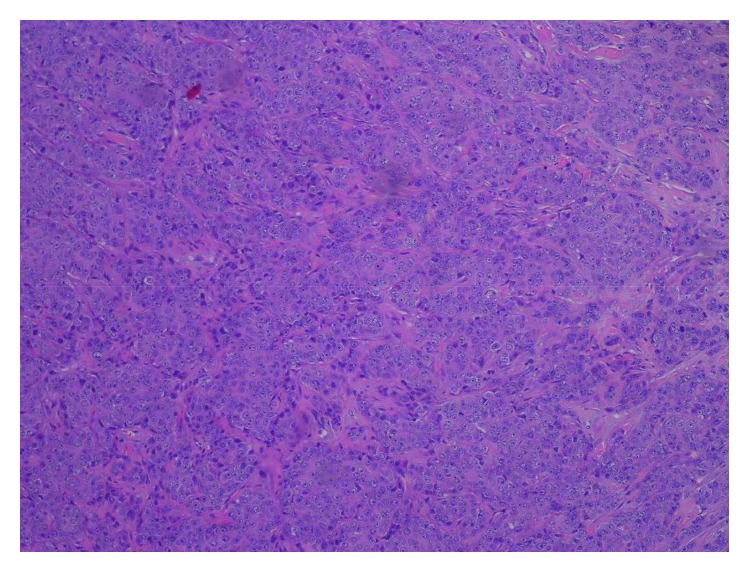
Eccrine carcinoma. Infiltrative, poorly differentiated neoplasm in a nested to trabecular pattern. Nuclei are relatively uniform with notably prominent nucleoli (hematoxylin-eosin, original magnification ×200).

She underwent wide surgical excision with clear margins attained, and three lymph nodes from the left inguinal zone were excised in April 2009. One lymph node was positive for disease involvement. A skin graft was placed thereafter. Histopathological analysis of the skin resection (Figure 1) supported the initial diagnosis of EC. Immunohistochemistry (IHC) resulted negative for the expression of estrogen and progesterone receptors and positive for HER-2/neu and EGF-R (2+ on 0–3+ scale). Albeit the aforementioned IHC data have been reported in EC, there are inconsistent and inadequate data in literature confirming it as a diagnostic tool. Unremarkable CT studies warranted no remaining disease.

**Figure 2:**
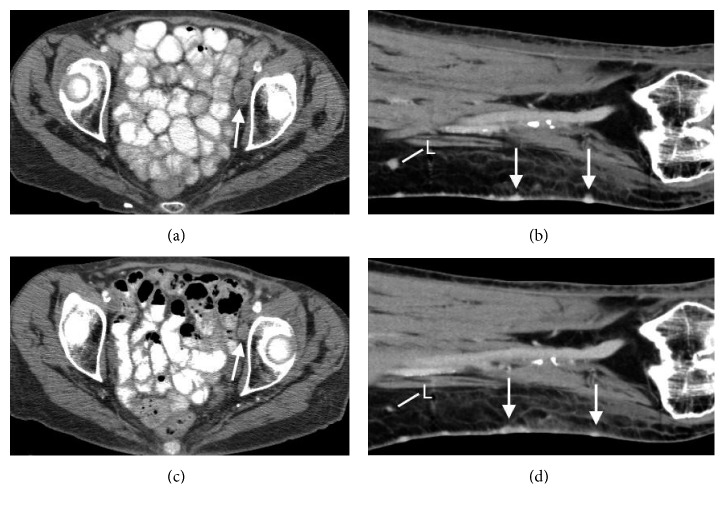
Progression of the disease despite treatment with carboplatin, paclitaxel, and radiotherapy (a, b), with subsequent partial treatment response two months after starting capecitabine (c, d). Axial postcontrast CTs of the pelvis show metastatic left pelvic lymph nodes, with decrease in size of a metastatic left external iliac chain lymph node from 2.2 × 1.1 cm (white arrow, a) to 1.5 × 0.9 cm (white arrow (c)) with capecitabine treatment. Coronal postcontrast CTs of the left leg (b) before and (d) after capecitabine treatment show similar cutaneous metastases (white arrows) with decreased size of a metastatic lymph node (L).

The patient returned in July 2009 with recurrent erythematous papular lesions throughout the skin graft of the left calf and new multifocal lesions in transit with the left medial thigh. A punch biopsy of the left thigh demonstrated recurrent metastatic EC. The metastatic lesion resulted negative for the aforementioned positive IHC stains on the primary tumor. CT studies indicated no internal organ metastasis. Surgical oncology performed a hypoxic-hyperthermic isolated limb perfusion on the left leg with melphalan and actinomycin D that resulted in a partial response. However, the patient returned in December 2009 with recurrent lesions along her left thigh and calf measuring 1-2 centimeters in diameter and appearing ulcerated. In March 2010, a regimen of intravenous carboplatin (AUC of 2) and paclitaxel (45 mg/m^2^) given on days 1, 8, and 15 (28-day cycle) concurrent to sensitizing 6 MV external beam radiation (4500 cGy over 25 fractions applied to the left extremity lesions) was performed, and she completed two cycles of therapy by May 2010. CT scans in October 2011 revealed left pelvic lymph node metastases (Figure 2(a)) as well as recurrence in the left leg (Figure 2(b)).

**Figure 3:**
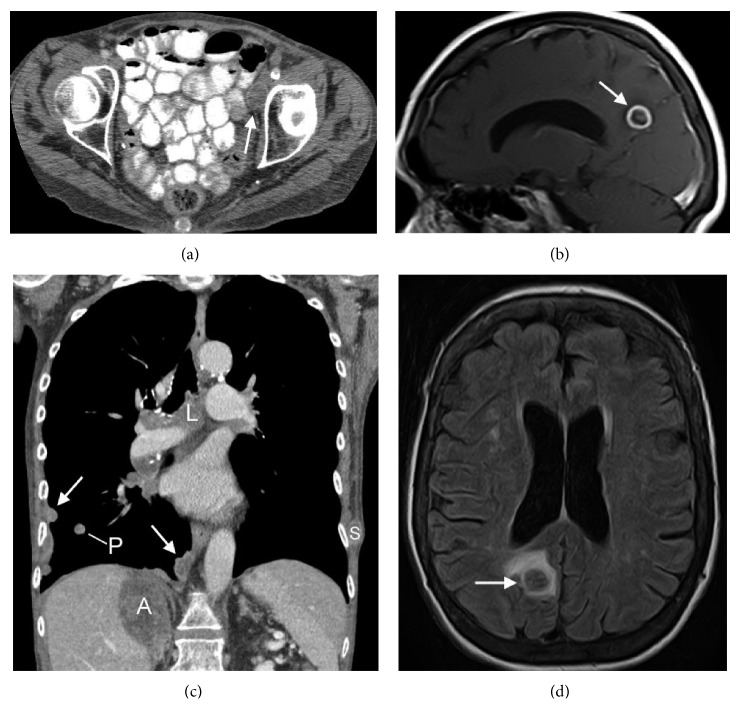
Progression of disease 18 months after starting capecitabine treatment. Axial postcontrast CT of the pelvis (a) shows increased size of a centrally necrotic left external chain lymph node, measuring 2.2 × 1.7 cm (white arrow). Postcontrast sagittal T1 MRI (b) and noncontrast coronal FLAIR MRI (d) of the brain show a new rim enhancing 1.2 cm metastasis in the right posterior parietal lobe with surrounding edema (white arrows). Coronal postcontrast CT of the chest and abdomen (c) shows new right pleural metastasis (arrows), mediastinal and hilar lymph node (L), and left lateral chest wall soft tissue (S) metastases, as well as an enlarging right lower lobe pulmonary metastasis (P) and right adrenal metastasis superimposed on an existing adrenal adenoma (A).

In February 2012, the patient started a capecitabine regimen (1500 mg PO q12h) that followed a 2-week on/1-week off dosing schedule. Due to grade 3 palmar-plantar erythrodysesthesia syndrome occurring 12 months after dosing, the dose was reduced to 1000 mg. The absence of cutaneous lesion recurrence was noted. CT studies (Figure 2) revealed an overall partial response with a decrease in size of the left pelvic lymph node metastases (Figure 2(c)) and cutaneous lesions in the left leg (Figure 2(d)). Capecitabine was continued until August 2013 when new cutaneous metastases appeared in the left gluteus (1.3 cm) and right anterior breast (1.7 cm). CT studies showed interval enlargement of left pelvic lymph node, right adrenal, and pulmonary metastases (Figures 3(a) and 3(c)). New metastatic disease was identified in the thoracic and abdominal lymph nodes, the right pleura, and cutaneous tissue (Figure 3(c)). An MRI of the brain revealed multiple metastases with index lesions in the right posterior parietal lobe (Figures 3(b) and 3(d)). The patient was referred to palliative care and hospice; she expired shortly after.

## 3. Discussion

Treatment strategies have been reported (Table 1) and illustrate the resistant nature of EC to numerous chemotherapies with only a few options increasing patient survival. Patients treated with platinum-based, taxane-based, and tyrosine kinase inhibitors seem not to derive a substantial therapeutic benefit. The tyrosine kinase inhibitor, sunitinib, achieved 8 months of PFS after a patient progressed through prior platinum-based therapy [10]. Poor outcomes are also noted with the anthracycline antibiotic, doxorubicin, and monotherapy with cyclophosphamide; EC rapidly progressed with metastases into multiple organs and the patients expired soon after [4, 11].

Distinct tumor markers have not been characterized for EC, and other authors have reported a variety of positive IHC stains [2, 6, 13]. Thus, correlative studies of biomarkers can help develop targeted therapies aimed at specific pathways and genetic mutations [2, 13]. For example, a case was reported of a patient with metastatic EC whose tumor biopsy revealed 100% reactivity for estrogen and progesterone receptors; the patient was started on empirical tamoxifen citrate therapy and achieved 3 years of complete remission [13]. On the other hand, our patient had negative reactivity for both of these receptors, so molecular profiles need to be identified and the prevalence assessed. A study that evaluated the molecular profiles of apocrine-eccrine carcinoma cases found that 6 of the 7 EC cases tested positive for the epidermal growth factor receptor (EGFR) mutant [14]. This highlights a potential role for clinical trials to determine the outcomes of targeted therapies that correspond to pertinent oncogene mutations [14].

To the best of our knowledge, we report the third case of metastatic EC treated with capecitabine [5, 12]. Our patient continued on capecitabine dosing from February 2012 to August 2013 without disease progression. Per RECIST version 1.1 criteria, our patient's follow-up CT study (Figures 2(c) and 2(d)) showed a partial response two months after starting capecitabine treatment. This regimen of oral capecitabine afforded efficacy and improved the patient's quality of life compared to the doublet therapy of carboplatin and paclitaxel which had more toxicities. The high mortality and morbidity rates associated with metastatic EC indicate the necessity for clinical trials to fully delineate safety and efficacy of potential future therapies [1–3, 9].

## 4. Conclusion

Eccrine carcinoma is an uncommon skin cancer that accompanies a poor prognosis. The clinical presentation of EC can be equivocal and result in misdiagnosis, which adversely affects patient outcomes. Standard of care treatment options for metastatic EC have not yet been developed. Further investigation into the efficacy of capecitabine treatment as monotherapy or in combination with other regimens for metastatic EC is worthwhile to improve the poor outcomes of diagnosed patients. Moreover, correlation of molecular profiles to tumor responses would provide helpful data to identify efficacious treatment regimens in the future.

## Figures and Tables

**Table 1:** Demographics, presentations, treatments, and outcomes of reported cases of metastatic EC.

Serial number	Age/sex [reference]	Presentation of primary cutaneous lesion	Metastatic sites	Treatment	Outcome
1	60/F [5]	Ulcerative, nodular, scaly, erythematous	Axillary and subclavicular LNs	Capecitabine	PFS (18 months)
2	73/M [12]	Ulcerative, nodular, erythematous	Parotid gland, cervical LNs, lung	Capecitabine	CR
3	64/F [13]	ND	Intraparotid LN	Tamoxifen	PFS (3 years)
4	43/F [10]	ND	Occipital LNs, cervical nerve root, vertebrae	Sunitinib	PFS (8 months)
5	45/F [11]	ND	Cervical LNs, bone, choroid	Doxorubicin	POD (deceased after 2 months)
6	59/F [4]	Bluish nodule, 2 cm diameter	Mediastinal LNs, lung	Cyclophosphamide	POD (deceased after 1 month)

CR: complete response; F: female; LN: lymph node; M: male; ND: not described; PFS: progression free survival; POD: progression of disease.
